# Taking Flight with Aviation English: How are Malaysian Teachers Preparing the Next Generation?

**DOI:** 10.12688/f1000research.169220.1

**Published:** 2025-10-03

**Authors:** AINA SURIANI MAHMOOD, Noor Saazai Mat Saad, Nurhayati Mohd Nur

**Affiliations:** 1 Universiti Kuala Lumpur, Dengkil, Selangor, 43900, Malaysia; 2 Universiti Sains Islam Malaysia, Nilai, Negeri Sembilan, Malaysia

**Keywords:** Aviation English, English teachers, Knowledge, Challenges, Pedagogy

## Abstract

**Background:**

Aviation English, a specialised language field for areas like aircraft maintenance, involves more complex pedagogy than general English, as it addresses specific learner needs within an aviation context. Teachers in this field must understand aviation-specific content and students’ linguistic needs to design courses that meet professional communication demands while adhering to legal and safety standards emphasizing clarity.

**Methods:**

This study examined Malaysian aviation English teachers’ experiences in developing tailored courses. Using semi-structured interviews with three teachers from aviation schools accredited by the Malaysian Qualifications Agency (MQA) and the Civil Aviation Authority of Malaysia (CAAM), data were transcribed and analysed in ATLAS.ti, with rigor ensured through member checking, prolonged engagement, and external audits.

**Results:**

Findings revealed three key themes: teachers’ understanding of aviation English, integration of aviation content, and instructional challenges. Participants described an extensive learning process in acquiring aviation knowledge and creatively integrating it into lessons through relevant activities and real-life applications. Challenges included varied student abilities, limited resources, and inadequate institutional support.

**Conclusions:**

The study explored how English teachers acquire and apply aviation knowledge and teaching skills, highlighting areas that could benefit from improved training and credentialing. Limitations include a small sample size and brief data collection period (March to August 2024), restricting generalisability, while the thematic analysis method may limit deeper exploration of nuanced experiences. Nonetheless, the study offers insights to enhance aviation English instruction for aviation students’ linguistic and professional needs.

## Introduction

Teaching English for Specific Purposes (ESP), particularly aviation English, is more challenging than teaching general English. As a specialised branch of ESP, Aviation English is tailored to students and professionals in aviation to support their communication in the workplace. ESP as a teaching approach is closely linked to curriculum design, teaching methods, and learning activities ( Hutchinson and Waters, 1987). The ESP model emphasizes three basic components: necessities, lacks, and wants ( Hutchinson and Waters, 1987). Hutchinson and Waters define “necessities” as the competences that students need to meet the demands of specific occupational contexts and that reflect what they will need to know in their future work environment. “Lacks” refer to the discrepancies between students’ current skills and the competences they need to acquire. “Wants” capture students’ personal learning goals or preferences. Identifying these different needs can guide the selection of resources and teaching activities and thus improve the overall quality of ESP teaching.

As language began to be understood differently depending on context, participants, and specific objectives, more emphasis was naturally placed on catering to learners’ needs and creating materials tailored to their specific requirements ( Jande & Ibrahim, 2021). This approach is in line with the ESP’s commitment to match teaching content to the practical and contextual needs of learners to ensure that materials and activities are focused on the particular skills needed in the professional environment. By focusing on relevant skills for the professional environment, ESP has evolved to develop resources that directly address the specific language needs of learners in their target areas. In this context, English language teaching should be linked to students’ academic and professional disciplines ( Mohamed et al., 2020). This approach ensures that students acquire not only general language skills, but also the specific vocabulary, formats and communication styles that are essential for success in their chosen disciplines.

However, a major challenge for English teachers is to find ESP approaches that successfully bridge the gap between English language teaching in the classroom and the practical language skills needed in the workplace ( Othman et al., 2017). These teachers face numerous demands from stakeholders who expect engineers to have competent language skills ( Othman et al., 2017). They also have to adapt to the rapid development of engineering education in the last two decades ( Bracaj, 2014). As a result, English teachers not only have the task of improving their students’ language skills but also of incorporating contemporary engineering knowledge and practices into their teaching methods. Bourni (2022) emphasises that expectations of ESP teachers in technical areas have become increasingly complex, largely due to rapid advances in industry. In addition, research suggests that ESP teachers are expected to manage an increasing workload while keeping pace with rapid advances in students’ fields of study. This combination contributes to teacher stress and limits the amount of time they can devote to developing curricula tailored to industry needs ( Bayram & Canaran, 2020). Another study discusses how increasing academic and administrative responsibilities leave little capacity for teachers to acquire the necessary technical skills, further impeding effective ESP teaching in specialised areas ( Kavanoz, 2020). These studies emphasise the double pressure that ESP teachers face: the need to teach specialised content that meets both the academic needs of students and the specific linguistic requirements of their future professions. In other words, English teachers are now tasked with not only teaching basic language skills but also integrating up-to-date engineering knowledge. This dual focus helps students develop language skills that are critical for both academic and professional success in technical fields.

Recognising the voices of English language teachers in the development of courses is therefore crucial, as their expertise is essential for the effective and sustainable design of these programs ( Rose and Galloway, 2019). Recent studies on ESP teaching show the importance of strengthening English teachers’ voices in course design and material selection to better meet students’ professional needs. Research by Basturkmen (2021) shows that ESP teachers play a crucial role in adapting materials and teaching approaches, so their insights are essential for addressing specific student needs. Furthermore, Lasagabaster (2022) underscores the need for ESP teachers to actively contribute their expertise to bridge the gaps between academic language teaching and real-life applications in specific professional fields, especially as the demand for domain-specific language skills increases. For countless years, this problem has been acknowledged as both cyclical and progressively worsening. However, most previous studies have not investigated specific domains, especially in aircraft maintenance area, but only conducted general studies on ESP ( Syamsinar and Jabu, 2016; Khamis et al., 2019; Meristo and López Arias, 2020; Rasyimah and Sari, 2018; Iswati and Triastuti, 2021). There is a lack of studies that take a comprehensive look at the entire career of English language teachers in ESP teaching and examine not only the challenges but also the path to success. Therefore, this paper examines the experiences of English teachers teaching English to aviation students, particularly in aircraft maintenance courses.

The guiding research question is: How do English teachers experience teaching English to aircraft maintenance students? This study adopts a comprehensive 360-degree approach from the teachers’ perspective and explores both the challenges and strategies. It explores their journey from an initial lack of aviation-specific knowledge to developing effective ESP teaching. The insights gained can help teachers and institutions develop solutions that better meet the needs of teachers and students in an aviation context.

## Literature review

### Importance of English for specific purposes in aviation

Communication skills are a crucial component for safety and efficiency in aviation. Yet English courses are often the only academic way to teach this essential skill as part of the undergraduate curriculum for aircraft maintenance students. These courses are important to provide students with the language skills they need to interpret technical manuals, articulate maintenance procedures and minimise communication errors. The ESP (English for Specific Purposes) programmes are specifically designed to develop the language skills required for aviation professionals in their specialised roles.

Estival and Molesworth (2020) highlight the importance of specialised language training in promoting effective communication between pilots, air traffic controllers and maintenance personnel and emphasise that tailored ESP curricula are crucial to meet the specific language needs of different aviation sectors. Similarly, Aiguo (2006) emphasises the need for integrated ESP curricula for aeronautics and aviation students that not only expand subject-specific vocabulary but also build the comprehensive language skills required for professional competence. In addition, Kicaj and Kicaj (2023) highlight the need for specialised language courses for aircraft mechanics, citing the lack of regulatory requirements for English proficiency as a contributing factor to the limited availability of such programmes, despite the significant role of English in aircraft maintenance. Meanwhile, the Civil Aviation Authority of Malaysia ( CAAM, 2021) has emphasised the importance of English language proficiency for aviation professionals and the need for special training programmes to overcome language barriers. The Malaysian Aviation Commission ( MAVCOM, *n.d*) has also emphasised the role of effective communication in ensuring safety and efficiency in the aviation sector. It has spoken out in favour of continuous professional development and the integration of English courses into aviation training. This emphasises the argument that targeted ESP training is essential for developing the communication skills that are vital for safety and operational efficiency in aviation.

### Challenges in ESP implementation

As Malaysia has positioned itself as a strategic hub in the Southeast Asian aviation sector, there is a growing demand for professionals who are proficient in English for Aviation, a specialised branch of English for Specific Purposes (ESP) tailored to the specific needs of the aviation sector. Aviation English goes beyond general English language skills and focuses on a precise and standardised use of language that is essential for safety and clear communication among aviation professionals, including pilots, air traffic controllers and maintenance technicians.

Teaching aviation English in Malaysia is a particular challenge, especially due to the lack of teachers who are familiar with both aviation and language teaching. Many English teachers lack the necessary aviation-specific training, which prevents them from organising lessons to meet the communication needs of the industry. Paramasivam (2013) states that the lack of aviation-specific language learning materials is a major challenge for practitioners. The study emphasises the importance of developing in-house training materials in collaboration with industry professionals to improve the English language proficiency of air traffic controllers in Malaysia. Furthermore, Mahmood et al., (2023) highlight that English teachers in Malaysian aviation schools face difficulties in designing aviation English materials, activities and curricula. These challenges are primarily due to a lack of textbooks and other aviation-specific resources. Similarly, Khamis et al., (2019) note that English teachers face limitations in teaching professional values, knowledge and understanding tailored to aviation. They also emphasise the shortcomings of teaching and learning English for Specific Purposes (ESP) in the aviation context.

These studies collectively underscore the need for a more robust framework for teacher training, resource development and collaboration with industry stakeholders to address the challenges of teaching aviation English in Malaysia.

### The delivery of ESP courses

English for Specific Purposes (ESP) courses are carefully designed to meet the specific language and communication needs of learners in different professional and academic fields. In the ASEAN region, these courses have played a key role in improving the English language skills of professionals and students, contributing to regional unity and economic growth.

The ESP curriculum aims to improve students’ communication skills by aligning them with industry requirements ( Singh, 2019). However, in Singh’s study, students’ performance was assessed only one month after the curriculum was introduced, which is a limited timeframe for assessment. Furthermore, the study lacks an in-depth exploration of the topic, limiting its scope and findings.

Numerous studies in the literature have emphasised the importance of conducting needs analyses to identify appropriate content for ESP modules or courses ( Iswati & Hastuti, 2021; Kaur, 2021; Niemiec, 2017; Petrus, 2014). These analyses highlight the need for curricula that are aligned with current industry requirements and take into account the rapid changes brought about by globalisation ( Lenard and Pintarić, 2018). As a result, universities are increasingly tasked with preparing graduates to meet global engineering standards, equipping them not only with technical expertise but also with essential non-technical skills such as communication. To meet industry expectations, academic institutions should work closely with employers to identify their needs and tailor English language courses accordingly. Engineering education researchers can further support these efforts by conducting comprehensive reviews of the current English curriculum to improve communication skills and assess whether graduates are meeting job requirements ( Wijesinghe & Jayawardane, 2019). Revising English courses for greater effectiveness remains an important priority.

Despite these efforts, the use of standard ESP textbooks often fails to address students’ specific needs for workplace communication ( Othman et al., 2017). In response, Othman et al. proposed a collaborative module developed by English and technical instructors at UKM, which reportedly improved participants’ language skills and confidence. The effective development of ESP curricula requires collaboration between students, educators and technical experts. Ye (2020) advocates analysing complex ESP contexts globally to identify appropriate teaching strategies. However, these studies lack broader applicability and do not include input from industry stakeholders on the mismatch between academic preparation and professional expectations.

Research into English teachers’ experiences of delivering ESP courses is crucial to understanding the challenges and opportunities associated with targeting language teaching to specific professional needs. The studies reviewed point to gaps in standard curricula, limited collaboration with industry stakeholders and a lack of customised teaching materials, all of which impact on the effectiveness of ESP courses. This study focuses on the experiences of English teachers and aims to provide practical insights into the realities of teaching ESP, particularly in aviation where accurate communication is crucial. Understanding teachers’ perspectives can make a valuable contribution to improving curriculum design, fostering collaboration between educators and industry professionals, and developing effective teaching strategies. This is in line with the overall aim of ensuring that ESP teaching prepares learners to fulfil the language requirements of their professions, thereby improving both individual skills and the efficiency of the sector.

## Research method

In order to explore the experiences of English teachers teaching aviation English at aviation schools in Malaysia, this study utilised semi-structured interviews, a method widely used in qualitative research. This approach is particularly effective in gaining detailed insights into specific phenomena as it allows for in-depth investigation with a limited number of participants ( Bloomberg & Volpe, 2008; Creswell, 2012). Qualitative research methods provide a nuanced understanding of a phenomenon by focusing on meanings, processes, and contextual factors, often exploring the “how” and “why” of particular issues or interactions ( Dworkin, 2012).

Participants were selected through purposive sampling to ensure relevance and expertise. Three English teachers currently teaching at aviation schools in Malaysia that are accredited by the Civil Aviation Authority of Malaysia (CAAM) participated in the study. Each had over ten years of teaching experience.

To maintain anonymity, participants’ names and institutional affiliations were not disclosed. Coded identifiers (T1, T2, and T3) were used, where “T” represents “Teacher.” T1 was a female teacher from Institution A with a PhD and 20 years of teaching experience, T2 was from Institution B with 10 years of experience, and T3 was from Institution C with 17 years of experience.

Ethical approval was obtained from the USIM Research Ethics Committee (Approval Ref: USIM/REC/2023/023). All participants received a participant information sheet and signed a written informed consent form prior to data collection. Participation was voluntary, with confidentiality ensured through anonymised data and coded references. The participant information sheet and consent form are available as extended data in Figshare [ 10.6084/m9.figshare.29886785.v1].

Semi-structured interviews lasting between 40 and 60 minutes were conducted to explore the teachers’ perspectives on teaching aviation English. It focused on the teachers’ experiences of teaching aviation English and provided an opportunity for them to share insights into their practices and challenges. The interviews were audio-recorded, transcribed verbatim, and returned to participants for member checking to ensure accuracy and clarity. The interview protocol used in the study is available as extended data in Figshare [ 10.6084/m9.figshare.30166033].

**Figure 1.**
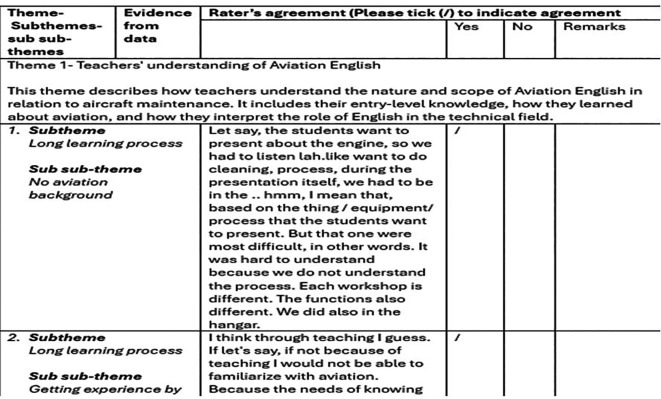
Sample from the external audit validation showing themes and supporting evidence.

To enhance the trustworthiness of the study, three strategies were employed. First, member checking was conducted by sending the interview transcripts to participants via email and, in some cases, through WhatsApp at their request. While only one participant responded with confirmation, the other two expressed their trust in the researcher and indicated that reviewing the transcripts was unnecessary. Second, prolonged engagement was established by initiating informal conversations and building rapport with each participant before the interview. This early engagement helped the participants feel more at ease and provided the researcher with deeper contextual understanding. Third, an external audit was carried out by an experienced ESP lecturer with over 15 years of experience in both maritime and engineering education, and with sufficient knowledge of the Malay language to interpret bilingual responses. She was provided with coded data, validation instructions, and an official letter of appointment. After reviewing the categorised themes and participant extracts, the expert indicated agreement with most of the themes. One disagreement was initially noted, but after a follow-up discussion, consensus was achieved. A sample of the external audit validation is provided in Figure 1. The full validation tables are available as Extended Data in Figshare [ https://doi.org/10.6084/m9.figshare.30166888.v1].

In this validation, the expert was asked to indicate her agreement or disagreement with the categorised themes corresponding to the participants’ extracts. She was instructed to tick “yes” if she agreed with a theme and “no” if she disagreed. In cases where there was a discrepancy between the coded data and the expert’s judgement, the researcher was responsible for addressing and resolving the issue through discussion. In this study, the expert agreed with all the categorisations that aligned with the participants’ extracts. Initially, one theme was questioned, but after discussion, the expert accepted the revised categorization.

These strategies collectively enhanced the credibility, dependability, and confirmability of the study. Data analysis followed Braun and Clarke’s (2006) six-step thematic analysis approach, using ATLAS.ti software to manage and organise the qualitative data effectively.

## Findings and Discussion

From the results findings, it shows that there are three main themes which related to the teachers’ experiences of teaching aviation English to aircraft maintenance students: teachers’ understanding of aviation English, integration of aviation content, and instructional challenges. The detailed breakdown of the themes and sub-themes is available as Extended Data (Table 1) ( https://doi.org/10.6084/m9.figshare.30198634.v1). The discussion in this section elaborates on the teachers’ journey from starting with no prior aviation knowledge, to applying content knowledge in English classes, and finally the challenges they encounter in teaching aviation English.

### Theme 1- Teachers’ understanding of aviation English

All the English teachers who participated in this study admitted that they went through a significant learning process that began with having no prior knowledge of aviation and eventually being able to teach basic aviation concepts to their students. The teachers pointed out that their academic backgrounds were in Teaching English as a Second Language (TESL) and art rather than aviation or specialized English programs. They further explained that they gained their knowledge of aircraft maintenance and aviation topics primarily through preparing and delivering lessons over time. They said like below:

*The process took longer and bit by bit in order to understand and prepare the materials for teaching.*      (T1)

*At first, I found difficult because I did not expect to teach at higher education university and to teach something related to ESP.*      (T3)

*I am not sure what to teach and how to teach. Then, I had to ask many people, then now I feel more confident.*      (T2)

The lengthy learning process seems to be an inherent aspect of teaching aviation English as it is outside the teachers’ area of expertise. Despite this challenge, the teachers have shown that they have succeeded in acquiring knowledge that goes beyond their actual area of specialisation. They reached a level of confidence where they felt able to teach the subject in the classroom despite having no prior knowledge.

For the second subtheme which named as sources of aviation knowledge, all teachers in this study reported that after being exposed to the teaching of aviation English at their respective institutions, they actively sought to expand their knowledge through various means. They consulted technical instructors, many of whom already had experience in the industry, accessed specialised books, internet resources and journals, and also sought advice and insights from their colleagues. T1, in her interview stated that she preferred to *“just go to the other technical instructors, they know the terms, the content, and how to say those tricky words properly. It’s just easier that way."* In their separate interviews, both T1 and T2 expressed similar views. They emphasised the importance of consulting technical instructors as they are more familiar with the technical terminology, technical content and correct pronunciation of aviation terms.

… *We’ve got the main department here, so if we need any resources, we just reach out to the aviation department instructors—they’re always willing to share with us.*      (T1)

*…I also contacted and asked some of the senior engineers who’ve been in the industry for quite a while. I’d ask them about the aviation jargon we should focus on teaching in class.* (T2).

In addition, the teachers made extensive use of technology to access information and utilised the ease and convenience of modern digital tools. T1 emphasised that today’s technological resources have significantly *helped ESP teachers compared to the past, especially in checking pronunciation and accessing relevant materials* (T1). Similarly, T2 acknowledged that she frequently relies on online resources and stated that she *continues to use them as an important part of her lesson preparation* (T2) *.* T1 also mentioned that she regularly c *ollaborates with colleagues who teach similar subjects* (T1) to discuss teaching materials and lesson topics. She emphasised the importance of reviewing her teaching after *‘postmortem’* to identify areas of improvement for her future teaching practise.

To summarise, the second sub-theme, Sources of Aviation Knowledge, highlights that English teachers act as part of a community of practise in which they actively seek support and guidance from technical instructors, colleagues and students. They also improve their understanding of aviation English through the use of books and modern technology. These collaborative interactions and shared experiences contribute to the creation of a collective knowledge base that fosters a deeper pool of resources and insights related to teaching English in an aviation context.

The last sub-theme assigned to teachers’ understanding of aviation English is entitled Requirements for teaching aviation English. Based on the interviews, all English teachers agreed that it is necessary to acquire at least a basic knowledge of aviation in order to teach the subject effectively. For example, T1 reported an experience where she could not properly evaluate a student’s presentation on a particular process or function of an instrument due to her limited knowledge, so she was not sure if it was valid. T3 and T2 similarly emphasised the importance of having basic aviation knowledge to effectively address such challenges. They shared their views as follows:

*…Like, why does the content have to be this way?*… (T2)

*…Honestly, you’ve gotta have at least some basic aviation knowledge, it is a must*… (T3)

All three participants agreed that aviation English teachers need an induction process to prepare effectively for teaching the subject. They emphasised the importance of adequate training to familiarise themselves with the content of aviation English courses and the methods and strategies for delivering them. This need arises from the fact that the people who are entrusted with teaching aviation English in their schools often have no prior knowledge or experience in the field of aviation.

The requirements for teaching aviation English are closely linked to the need to have a basic knowledge of the subject and to receive appropriate training. Such training would provide teachers with effective methods and strategies for the delivery of aviation English courses. Furthermore, this sub-theme is closely related to the third theme, Challenges in teaching aviation English, and illustrates how these aspects are interlinked when it comes to managing the complexity of teaching in this particular field.

In this study, the English teachers began teaching aviation English without prior knowledge of the subject, as it is not their primary specialism. For these teachers, teaching aviation English is not a one-time task, but rather an ongoing process that evolves with their ongoing assignments and interactions with students. Gaining an understanding of aviation concepts and integrating them effectively into the classroom requires a prolonged and iterative learning process that reflects the complexity of mastering and applying this specialized knowledge.

The results of this study are consistent with previous research by Estival and Molesworth (2020), which emphasises the importance of dedicated language training for effective communication between pilots, air traffic controllers and maintenance staff. Their study emphasises the need for tailored ESP curricula to meet the specific language requirements of different aviation sectors. The Civil Aviation Authority of Malaysia (CAAM) has also highlighted the critical role of English language skills for aviation professionals and the need for specialized training programmes to overcome language challenges.

Furthermore, the uncertainty expressed by the participants in this study about whether their teaching matched the needs of the industry reflects the findings of another research. For example, Othman et al. (2017) found that English language instructors often experience confusion in identifying ESP practises that effectively bridge the gap between classroom instruction and workplace language use. This indicates a persistent gap between professional discourse and ESP teaching, which complicates the development of relevant and practical language instruction.

In addition, recent studies confirm that despite undergoing English or ESP training, many employees and trainees continue to face difficulties in applying their language skills in actual work settings. More specifically, these difficulties are often related to grammar, pronunciation, understanding different accents, and using appropriate vocabulary or idiomatic expressions, which are crucial for effective workplace communication and career advancement ( Glomo-Narzoles & Glomo-Palermo, 2021; Singh & Harun, 2020; Putra et al., 2022; Liang et al., 2025).

Moreover, English language teachers are under increasing pressure from stakeholders who expect graduates, particularly in engineering and aviation fields, to demonstrate a high level of English fluency ( Othman et al., 2017). Taken together, these findings underscore the urgent need for closer collaboration between educators and industry stakeholders to ensure that ESP courses are meaningfully aligned with workplace communication requirements.

### Theme 2 - Integration of aviation content

The discussion shows that English teachers lacked specialised training in aviation English and relied on discussions with colleagues, industry-experienced technical instructors, and online resources for content and pedagogy. The next section explores classroom implementation, focusing on activities and materials that integrate technical aspects and real-world applications.

As there is no specific textbook for teaching aircraft maintenance, teachers have taken the initiative to develop their own material. T1 explained,

*…There is no specific textbook for this, so I had to create my own notes. It’s not easy — I went to the trouble of creating the slides and notes before printing them out for the students…*      (T1)

T3 expressed a similar view,

*…We don’t have a textbook, just the syllabus. So, I collect and create the materials myself based on that….*

She also emphasised *,*

*…I like my students to work together — lots of collaborative learning, pair work, group tasks — it helps them to be more engaged*…      (T3)

All teachers who took part in the study emphasised the use of collaborative learning activities in their teaching. They agreed that group tasks are particularly effective as students preferred working together. T2 shared,

*…I usually have them work in groups, especially on writing assignments. This helps them to discuss and share ideas…*      (T2)

She added that she likes to *encourage them to have in-class discussions about certain topics — it gets them thinking and talking.*      (T2)

This collaborative approach reflects the teachers’ efforts to encourage teamwork and active student engagement. In addition, participants agreed that they like to use interactive learning in the classroom to follow the current trend towards technology-intensive environments. They said like below.

*…I love showing them videos—it really helps the students visualize things better when I use that approach…*      (T1)

*…I actually don’t encourage them to use slides for presentations. I prefer they use a canvas instead—it’s way more interactive…*      (T3)

*…For me, I like using Kahoot. It’s just more fun compared to other tools…*      (T2)

Overall, despite limited knowledge and resources, English teachers at these aviation schools showed motivation and effort in preparing activities, materials and assessments for their classes. They sought information in various ways, such as consulting technical instructors and using online resources to tailor their lessons to meet the needs of their students. However, they are still unsure whether their preparations are correct and appropriate.

For the second sub-theme, integrated with the technical part, all participants emphasised that they take an integrative approach to combine general topics with technical content relevant to their students’ backgrounds. T3 shared that she tailors assignments and presentations to students’ fields of study and emphasised that *when the lessons connect to their field, they are more engaged and show interest* (T3). Similarly, T2 explained *“I know that I need to include the technical vocabulary and jargon in my lessons”* (T2). She went on to explain that she often links her activities to the licensing module her students are studying that week, saying *“I try to keep the activities light as they have already covered the heavier technical topics in their other courses”* (T2). T1 noted that even with general topics such as memo writing, emails or job search skills, she ensures that the lessons and activities are tailored to the students’ areas of expertise.

Overall, the integration of technical elements into aviation English courses goes beyond simply combining general English and aviation-related content. It is also about aligning lessons with the current topics students are learning in their technical courses to provide a cohesive and parallel learning experience.

In the final sub-theme, *real-life learning,* participants emphasised their efforts to familiarise students with real-world problems in an aviation context. One participant shared her experience of *taking the students to the hangar and workshop where they were asked to create a presentation based on their observations* (T1). Similarly, T3 and T2 described their approaches:

*…I have done innovation projects where students use recycled materials to create something useful for their field…*      (T1)

*…I take the students to the library. For example, when we are looking at ATA for wings, I let them find the information on their own — it helps them engage with the content…*      (T2)

As T1 and T2 noted, bringing real-world materials and environments into the classroom allows students to relate new concepts and ideas to their own experiences, which promotes deeper understanding. T3’s use of project-based learning (PBL) is an example of a strategy that allows students to develop their knowledge and skills through engaging projects based on real-world challenges. These activities represent innovative approaches used by English teachers at the three aviation schools. However, the teachers acknowledged that such methods cannot be implemented in every English courses due to constraints such as limited time and resources, which will be discussed in the following section.

Overall, three sub-themes were identified under the broader sub-theme of Implementation of Content Knowledge: Activities and Material Preparation, Integration with technical content, and Real-Life Applications. These sub-themes outline the approaches English teachers use to implement English aviation content in the classroom, including what is taught and how it is taught. The findings suggest that teachers spend considerable time researching teaching materials, methods, and activities to address the specific needs of their students. However, despite these dedicated efforts to ensure lesson effectiveness and smooth delivery, they continue to face persistent challenges. Among the most reported issues are the pressure of managing extensive syllabi, overcrowded classrooms, demanding assessment requirements, and limited access to instructional materials. When these constraints occur simultaneously, they can seriously limit teachers’ ability to respond effectively to individual student needs ( Gulab et al., 2024; Yonas et al., 2023).

A key aspect of effective implementation involves the integration of language instruction with technical content in ways that mirror real-world aviation contexts. In this regard, real-life application is seen as essential in bridging the gap between theoretical knowledge and practical use. Herrington et al. (2003) have proposed ten characteristics of authentic activities or tasks that provide essential conditions for bridging the gap between classroom learning and real-world application. Among these features, connection to the real world is a key factor. This refers to learning experiences that are directly related to students’ aspirations, backgrounds, interests or real-world problems and challenges. When technical concepts are taught through contextualized and relatable scenarios, such as budgeting, scheduling, or managing technical documents, students become more engaged and motivated, as they can see the practical relevance of what they are learning. These types of tasks promote higher-order thinking and support the development of essential life and workplace skills ( Antao & Morales, 2025).

Further enhancing the authenticity of instruction, teachers also incorporate real-world materials into the classroom environment. To achieve this, they often utilise *realia*, which refers to real-life objects, documents, and materials that provide tangible connections to professional practices and cultural contexts. Realia supports students in understanding how language operates within the aviation industry and across diverse societies. Studies have shown that the use of realia enriches classroom experiences, making lessons more interactive, culturally grounded, and memorable ( Argawati, 2009; Lee et al., 2021; Sen & Ray, 2021; Ibad & Sarifah, 2021). Beyond supporting language acquisition, realia also foster intercultural awareness, as students engage with materials that reflect authentic social and workplace practices ( Aziza, 2024; De La Red, 2022; Javorcikova & Zelenkova, 2019).

As a whole, the integration of authentic activities, technical content, and real-life resources represents a pedagogical approach that not only reinforces communication skills but also prepares students to meet the expectations of the aviation workplace. However, the effectiveness of these approaches depends largely on institutional support, adequate resources, and manageable teaching conditions, all of which remain ongoing challenges for many educators.

### Theme 3- Instructional challenges

The challenges of teaching aviation English to aircraft maintenance students can be categorised into four sub-themes: the nature of aviation English learning, student skills and attitudes, inadequate resources and institutional support. These challenges are detailed below.

All participants acknowledged that they needed more time to understand the aviation context and apply it effectively in their teaching. As they had no experience in aviation background, they initially found it difficult to understand the technical topics they needed to incorporate into their lessons. The teachers admitted that they had difficulty pronouncing technical jargon, understanding aviation-related articles and grasping the meaning of technical terms. T1, for example, shared her experience:

*…In the beginning, it was quite difficult to figure out how to pronounce the technical terms myself. I often got it wrong because I wasn’t familiar with words or terminology…* (T1)

In addition to the problems with pronunciation, T1, admitted that she knew little about certain subjects and technical terms. She sometimes had to appear to understand these concepts, especially during student presentations. T3 and T2 also commented that they often needed extra time to understand aviation-related content, especially when teaching jargon.

Teaching and learning aviation English were particularly challenging as the teachers were not initially familiar with the field. They had to go through a lengthy learning process to master the aviation context and integrate it into their teaching. This slow pace was compounded by the different language levels of the students, which led them to adopt a “ *trial and error*” approach to their teaching methods.

The English teachers also pointed out the challenges posed by the different educational backgrounds of the students and the different levels of interest in teaching English. Not all students had a technical background, so teachers like T2 had to spend extra time to motivate them and start with the basics. She addressed this problem by encouraging group discussions to promote knowledge sharing. However, some *students saw English as less important than technical subjects* (T2) and considered communication skills unnecessary for technical careers. This attitude hindered engagement, especially in written activities.

T2 noted that students often *questioned the necessity of writing essays or improving communication skills* (T2), while T3 observed that students *struggled with grammatical accuracy* (T3), particularly with the use of the passive voice in technical writing tasks. She shared that student often made mistakes in process descriptions and favoured the active form despite being instructed to use the passive voice.

It was clear from the interviews that students’ skills and attitudes significantly impacted the teaching experience and contributed to slower progress in aviation English lessons. Whilst such challenges are common, they also highlight areas where improvements could enhance the learning experience.

Given their limited knowledge of aviation, all participants reported that they had made considerable efforts to acquire the necessary expertise to make their courses run smoothly. However, they also highlighted challenges arising from insufficient resources to acquire this knowledge.

T1 pointed out that there are *no specific textbooks for teaching aircraft maintenance* (T1), unlike the resources available for teaching English to pilots or air traffic controllers. Similarly, T2 highlighted the prohibitive cost of aviation-related textbooks, which was beyond her budget. To work around these limitations, she relied on *consulting technical instructors, exchanging ideas with colleagues, and using online resources* (T2) to gather the information she needed.

T3 also pointed out the problem of limited teaching material and explained that she could only find one relevant book at a book fair. However, she noted:

*…It’s English for aviation, not specifically for aircraft maintenance, so it doesn’t quite meet my needs…*      (T3)

Unlike English textbooks designed for pilots or air traffic controllers, resources specifically tailored to aircraft maintenance are far less accessible. Due to this lack of materials, English teachers do not have sufficient information to plan and deliver their lessons effectively, which adds to the challenges of teaching aviation English.

The final sub-theme of challenges in teaching specialised English highlights inadequate institutional support, including heavy workloads, rigid syllabus and inadequate training of English teachers. These challenges have a significant impact on teachers’ ability to teach English effectively in aviation.

Teachers reported that the excessive amount of teaching hours leaves little time for lesson preparation. T2 described having six hours of classes a day and having no choice but to repeat activities due to time constraints. She expressed frustration at having to teach English for multiple programs which sometimes led to confusion and errors in content delivery.

*“With so many classes, I am just preparing what I already have. At one point, I had 18 credit hours a week — six hours a day. There was no time to revise anything or develop new activities*," she said.

T1 expressed similar concerns, noting that her current tight schedule limits her ability to go on field trips or spend time in the hangar and workshop with students, activities she used to organise.

Another challenge was the lack of flexibility in the prescribed syllabus. T3 mentioned that she strictly adhered to the syllabus as it was mandatory and was developed by the curriculum department based on the needs of the industry. In contrast, T1 had the opportunity to collaborate with her colleagues in developing the English syllabus for her school.

“ *We have to follow the curriculum — it’s compulsory. It’s supposedly based on a needs analysis, but we are not involved in creating it*," T3 explained.

The teachers also pointed out the inadequate training in teaching aviation English. T3 acknowledged that her limited aviation knowledge prevented them from effectively preparing students to communicate in the workplace. She expressed the need for specific training:

*"If I had some basic knowledge of aviation, I could prepare my students better. Right now, it’s mostly general English that’s not so applicable to their work.*"      (T3)

Inadequate institutional support compounds the challenges faced by English language teachers in teaching aviation English, along with the sub-themes of limited resources, student skills and attitudes, and the nature of aviation English learning. Without sufficient aviation knowledge, customised training and up-to-date resources, teachers consistently struggle to meet the demands of the subject. Addressing these systemic issues is critical to improving the quality and effectiveness of aviation English teaching.

The findings of this study are consistent with previous research highlighting the challenges faced by English language teachers when teaching specialised subjects. Othman et al. (2017) found that stakeholders expect engineers to be fluent in English, which requires educators to keep up with advances in engineering education, such as new technologies, teaching methods, and interdisciplinary approaches ( Bracaj, 2014). In line with this, the current study reveals that English teachers face additional challenges due to their limited expertise in aviation, similar to the findings of Khamis et al. (2019) who observed a lack of professional orientation among ESP teachers in specialized fields. Furthermore, the growing emphasis on English proficiency and communication skills as core hiring criteria among employers ( Mohamed et al., 2020) reinforces the need for graduates who can function effectively in international and multicultural work environments.

Similarly, the results are consistent with those of Yusof et al. (2017) who identified issues such as difficulties in implementing the curriculum, student-related concerns, lack of time, limited resources and lack of training which, as Iswati and Triastuti (2021) noted, are often due to inadequate preparation for curriculum change that impacts teaching practice. As a result, English language teachers are under pressure to demonstrate their competence and intellectual ability, as Melles et al. (2005) emphasise. These interlinked challenges emphasise the need for targeted support and training for teachers in specialist areas such as aviation English.

Overcoming these issues depends on consistent work in developing teaching methods and professional expertise. To summarise, ESP teachers’ commitment to contextual understanding, continuous professional development and the delivery of relevant content and tasks provides a solid foundation for improving the quality of English language teaching. These efforts enable students to excel in their respective disciplines while fostering effective communication in both professional and academic contexts.

Although the study involved only three aviation English teachers in Malaysia, the findings have relevance for other ESP contexts where educators teach technical subjects without prior domain expertise. The strategies identified, such as collaborating with industry practitioners and creating authentic, field-specific materials, can be adapted to fields like maritime, engineering, and medical English. By focusing on teachers’ learning and adaptation processes, this study adds a less explored dimension to ESP scholarship, offering practical insights for teacher development and curriculum planning. The credibility of the findings was reinforced through member checking, prolonged engagement, and an external audit, with systematic thematic analysis and rich contextual descriptions supporting their dependability, confirmability, and transferability.

## Conclusion

In summary, English teachers in aviation institutions have undergone a significant learning process to acquire knowledge of aviation English, an area outside their specialism. In the interviews, they talked about the sources of aviation knowledge, the requirements for teaching, and the development of activities and materials to equip students with industry-relevant language skills. Although they have been successful in delivering courses, teachers face ongoing challenges as their responsibilities extend beyond teaching to mastering a subject area in which they had no prior experience.

### Limitation & Further research

The limitations of this study include the small sample size and the relatively short period of data collection (March to August 2024), which involved only three English teachers teaching at aviation institutions in Malaysia, limiting the generalisability of the results. Furthermore, while the method of thematic analysis provided valuable insights, it may have prevented a deeper exploration of the nuanced experiences of the participants. Despite these limitations, the study offers critical perspectives for the improvement of English language teaching in aviation, addressing both linguistic and professional needs.

Meanwhile, the limited amount of research on aviation English, particularly in the context of aircraft maintenance, emphasises the study’s potential as a foundation for future research. The findings point to several areas that require further research beyond the delivery of ESP courses. Future studies should investigate the factors contributing to the lack of communication skills of aircraft maintenance technicians and engineers, including the prioritisation of passing exams over acquiring practical skills and the effectiveness of English courses offered in their schools.

In addition, research should focus on ensuring lifelong learning and the sustainable use of English communication skills for students and professionals in the aviation industry. The challenges identified in this study, especially those highlighted in Theme 3, such as inadequate resources, the lack of specialized training and the heavy workload of teachers, also require institutional attention and further investigation. Addressing these issues will be instrumental in improving the quality of English language teaching in aviation and meeting the evolving needs of the industry.

## Data Availability

Figshare: Anonymised interview transcripts for the study *[Taking Flight with Aviation English: How are Malaysian Teachers Preparing the Next Generation?]* https://doi.org/10.6084/m9.figshare.29886785 This project contains the following underlying data:
• T1_INTERVIEW (English teacher from Institution A) • T2_INTERVIEW (English teacher from Institution B) • T3_INTERVIEW (English teacher from Institution C) Figshare: External audit validation table [ https://doi.org/10.6084/m9.figshare.30166888.v1] Figshare: Table 1: Findings for Teachers’ experiences [( https://doi.org/10.6084/m9.figshare.30198634.v1] Figshare: Plain Language Statement [ https://doi.org/10.6084/m9.figshare.30166774.v1] Figshare: Consent form [ https://doi.org/10.6084/m9.figshare.30166732.v1] Figshare: Interview protocol [ https://doi.org/10.6084/m9.figshare.30166033.v1] Data are available under the terms of the Creative Commons Attribution 4.0 International license (CC-BY 4.0).
