# Supplementary material for: Taking Flight with Aviation English: How are Malaysian Teachers Preparing the Next Generation?
Source: F1000Res. 2025 Oct 3;14:1036. [Version 1] doi: 10.12688/f1000research.169220.1 (PMC12569509; doi:10.12688/f1000research.169220.1)
Supplement: Peer review history

# Reviewer report: Reviewer response for version 1

**DOI:** 10.5256/f1000research.186515.r420965

**Published:** 2025-10-28

**Authors:** Anna Borowska

**Affiliations:** 1 University of Warsaw, Warsaw, Poland

The article seems to be a basic one and it does not refer to important sources for Aviation English training /teaching nor the training of trainers. It is recommended to refer to well-known literature, e.g. Anna Borowska Avialinguistics and others. Thera are no references to ICAO and their recommendations for Aviation English Course delivery. 

 Author should also characterise an aviation English trainer and rules they have to follow, e.g. Supporting Aviation English Trainers and Assessors.

 It can be indexed provided the author introduces corrections.

Is the work clearly and accurately presented and does it cite the current literature?

Partly

If applicable, is the statistical analysis and its interpretation appropriate?

Partly

Are all the source data underlying the results available to ensure full reproducibility?

Partly

Is the study design appropriate and is the work technically sound?

Partly

Are the conclusions drawn adequately supported by the results?

Partly

Are sufficient details of methods and analysis provided to allow replication by others?

Partly

Reviewer Expertise:

Avialinguistics, Aviation English, aeronautical Communication, teaching Aviation English

I confirm that I have read this submission and believe that I have an appropriate level of expertise to confirm that it is of an acceptable scientific standard, however I have significant reservations, as outlined above.

# Reviewer report: Reviewer response for version 1

**DOI:** 10.5256/f1000research.186515.r420968

**Published:** 2025-10-22

**Authors:** MALILA PRADO

**Affiliations:** 1 Beijing Normal University & Hong Kong Baptist University United International College, Zhuhai, China

The topic is timely and relevant, especially within the under-researched area of English for aircraft maintenance. Particularly, it is a valuable study in terms of its discussion. The authors present thoughtful considerations into the realities of English teaching within this specific context. Even though the study involves only three participants, the depth and quality of the teachers’ responses demonstrate their professionalism and strong commitment to their work. Their awareness of the challenges they face and their willingness to adapt their practices show that these are experienced and reflective practitioners.

 Additionally, addressing teachers in such a highly technical environment can also benefit the readers of the journal. Their findings can assist in setting the scene to other studies.

 However, it would strengthen the paper if the authors included brief information about the participants’ professional backgrounds, such as their years of experience in aviation English or in higher education. The paper notes that all participants are English majors that needed to learn the specificity of aviation while in service, but how long did it take them to feel more comfortable? Such information is important in our field in which English language teachers often enter aviation contexts with limited domain knowledge at first and later develop professional expertise through practice and collaboration (see ICAO’s Doc 9835 and Circular 323).

 This would also be a good opportunity for the authors to acknowledge a broader question within ESP teacher development: Who is the most effective English teacher in highly specialized fields: an industry expert with language training, or an English language teacher who progressively learns to handle domain-specific needs?

 Additionally, while the discussion section is strong, the introduction and literature review could be narrowed. At present, they tend to circle around the same points, particularly the idea that ESP is “more challenging than general English” and that teachers “must bridge the gap between English language teaching and real-world language needs”. These claims appear many times in different wording, creating unnecessary repetition. Rather than emphasizing “difficulty” or “double pressure”, the authors might reframe ESP as contextually different. General English and ESP can both be demanding in different ways; the difference is that ESP requires a more targeted understanding of communicative practices and genres.

 Regarding the paper’s conceptual clarity, there needs to be an explanation of what the authors understand as aviation English from start. It is an umbrella term that covers many different professionals and genres, although it is more commonly understood as radio communication between pilots and controllers. But this paper shows that this is not always the case.

 On page 4, the authors say:

 “Aviation English goes beyond general English language skills and focuses on a precise and standardised use of language that is essential for safety and clear communication among aviation professionals, including pilots, air traffic controllers and maintenance technicians.”

 Precisely – but this should go from the beginning. I also suggest including this scope along with references to the few yet important studies on it:

 Shawcross (1992) for textbook

 (Chatzi et al., 2019; Drury & Ma, 2003; Dupont, 1997; Ma et al., 2009) – for maintenance errors and safety issues

 (Embryany & Ratmanida, 2020; Terenzi, 2021; Terenzi & Pizzi, 2020) for aircraft maintenance and English teaching

 Including these references will assist in covering the connection between English for maintenance and safety (particularly through documentation), in acknowledging the distinct genres of maintenance documentation, manuals and task cards, and in specifying the scope.

 The quote from a study participant on p. 11 illustrates this point:

 “…It’s English for aviation, not specifically for aircraft maintenance, so it doesn’t quite meet my needs…”

 This shows that these are considered two different terms.

 As for the conceptualization of ESP, although correct, relaying largely on Hutchinson and Waters (1987) (needs, lacks and wants) will make this study sound incomplete. The authors can include more recent developments in ESP and, for example, genre-based pedagogy (Bhatia), or technology-based pedagogy (Paltridge, Charles). The authors touch on many important aspects in the Discussion session, but they may take the reader off guard without previous preparation. Aspects such as community, agency, professional discourse, situated practices, learner agency, among others, are all taken into consideration in latest scholarship.

 As I mentioned earlier, this is a much-needed study, and its strongest point is the discussion section. I suggest reviewing the introduction and the literature to highlight its significance.

Is the work clearly and accurately presented and does it cite the current literature?

Partly

If applicable, is the statistical analysis and its interpretation appropriate?

Not applicable

Are all the source data underlying the results available to ensure full reproducibility?

Yes

Is the study design appropriate and is the work technically sound?

Yes

Are the conclusions drawn adequately supported by the results?

Yes

Are sufficient details of methods and analysis provided to allow replication by others?

Yes

Reviewer Expertise:

Aviation English, aviation English teaching, material design, corpus linguistics, pragmatics.

I confirm that I have read this submission and believe that I have an appropriate level of expertise to confirm that it is of an acceptable scientific standard, however I have significant reservations, as outlined above.

## References

[1] : The Unexplored Link between Communication and Trust in Aviation Maintenance Practice. *Aerospace* .2019;6(6) : 10.3390/aerospace6060066 10.3390/aerospace6060066

[2] : Language Error in Aviation Maintenance: Quantifying the Issues and Interventions in Four World Regions. *The International Journal of Aviation Psychology* .2009;20(1) : 10.1080/10508410903416136 25-47 10.1080/10508410903416136

[3] : Overcoming Challenges in English for Aviation Maintenance: Technical Publications Selection for the Construction of a Corpus and Its Use to Teach Language Aspects Considering Learners Needs. *Open Journal of Applied Sciences* .2021;11(10) : 10.4236/ojapps.2021.1110084 1122-1134 10.4236/ojapps.2021.1110084

[4] : The relevance of linguistic and professional knowledge as contributing factors for technical translation in the area of aircraft (maintenance). *The Especialist* .2020;41(3) : 10.23925/2318-7115.2020v41i3a9 10.23925/2318-7115.2020v41i3a9

[5] : Do Language Barriers Result in Aviation Maintenance Errors?. *Proceedings of the Human Factors and Ergonomics Society Annual Meeting* .2003;47(1) : 10.1177/154193120304700110 46-50 10.1177/154193120304700110

# Reviewer report: Reviewer response for version 1

**DOI:** 10.5256/f1000research.186515.r420962

**Published:** 2025-10-09

**Authors:** Laila Rochmawati

**Affiliations:** 1 Politeknik Penerbangan Surabaya, Surabaya, Indonesia

Evaluation Summary of Aviation English Study

 1. Clarity and Accuracy of Presentation

**Answer**: Yes

 The study is clearly and accurately presented with a well-structured format, including sections like abstract, introduction, literature review, methods, findings, discussion, and conclusions. The language is precise, accurate, and accessible. It cites a balanced mix of foundational literature (e.g., Hutchinson & Waters, 1987) and recent studies up to 2023 (e.g., Mahmood et al., 2023; Kicaj & Kicaj, 2023), covering key topics in ESP and aviation English. Citations are relevant and support arguments without notable gaps.

**Evidence**:

- Page 3: "The ESP model emphasizes three basic components: necessities, lacks, and wants (Hutchinson and Waters, 1987)."
- Page 4: "Estival and Molesworth (2020) highlight the importance of specialised language training..."

**Suggestions for Improvement**:

- Rephrase page 3 to: "The ESP model, as defined by Hutchinson and Waters (1987), emphasizes three fundamental components: necessities, lacks, and wants."
- On page 4, add "as evidenced in their experimental study" after Estival and Molesworth (2020).
- On page 8, expand to: "The results align with Estival and Molesworth’s (2020) findings from an experimental study on communication errors..."
- Include 2024–2025 citations on post-pandemic ESP adaptations in aviation (pages 14–15).

2. Appropriateness and Technical Soundness of Study Design

**Answer**: Yes

 The qualitative design, using semi-structured interviews and thematic analysis (Braun & Clarke, 2006), is appropriate for exploring teachers’ lived experiences in aviation English. The small sample (n=3) is justified for in-depth, purposive sampling in exploratory research. Technical soundness is shown through ATLAS.ti analysis and trustworthiness measures (e.g., member checking, prolonged engagement, external audit). Ethical considerations, including USIM ethics approval and anonymity, are addressed.

**Evidence**:

- Page 5: "This study utilised semi-structured interviews, a method widely used in qualitative research."
- Page 5: "Participants were selected through purposive sampling..."
- Page 6: "To enhance the trustworthiness of the study, three strategies were employed..."

**Suggestions for Improvement**:

- Rephrase page 5 to: "This study employed semi-structured interviews, a widely recognized method in qualitative research (Bloomberg & Volpe, 2008; Creswell, 2012)."
- On page 6, expand to: "These strategies—member checking, prolonged engagement, and external audit—enhanced credibility, dependability, and confirmability."
- On page 13, rephrase limitation to: "While the small sample (n=3) enables rich insights, it limits generalizability, as per Creswell (2012); future research could use mixed methods."

3. Sufficiency of Methods and Analysis Details for Replication

**Answer**: Yes

 The methods section details participant selection, 40–60-minute audio-recorded interviews, six-step thematic analysis in ATLAS.ti, and trustworthiness strategies. Extended data (e.g., interview protocol, consent forms) are available via Figshare DOIs, enabling replication. Sufficient quotes and procedural descriptions are provided despite withheld transcripts for anonymity.

**Evidence**:

- Page 5: "Semi-structured interviews lasting between 40 and 60 minutes were conducted..."
- Page 6: "Data analysis followed Braun and Clarke’s (2006) six-step thematic analysis approach..."

**Suggestions for Improvement**:

- On page 5, add: "Interviews were conducted virtually or in-person based on participant preference."
- Rephrase page 6 to: "Data were analyzed using Braun and Clarke’s (2006) framework within ATLAS.ti, facilitating coding and theme development."
- For Figure 1 (page 6), add caption: "Excerpt from external audit validation table (full table via Figshare DOI)."
- On page 13, note: "Anonymized transcripts ensure ethical standards; ATLAS.ti project file available upon ethical review."

4. Applicability of Statistical Analysis

**Answer**: Not applicable

 This is a qualitative study with no quantitative data or statistical analysis.

 5. Availability of Source Data for Reproducibility

**Answer**: Yes

 Extended data, including participant information sheets, consent forms, and validation tables, are accessible via Figshare DOIs, ensuring reproducibility. Raw elements like quotes and themes are sufficient for qualitative work.

**Evidence**:

- Page 13: "Figshare: Anonymised interview transcripts... [DOI: 10.6084/m9.figshare.29886785]."

**Suggestions for Improvement**:

- On page 13, add: "Pseudonyms (T1, T2, T3) protect confidentiality; ATLAS.ti project file available upon ethical review."
- Ensure DOIs are hyperlinked in the final PDF.

6. Support for Conclusions by Results

**Answer**: Yes

 Conclusions reflect three themes: teachers’ understanding of aviation English, content integration, and instructional challenges. Limitations (small sample, short data collection period) are acknowledged, and practical implications (e.g., improved training) are suggested without overgeneralization.

**Evidence**:

- Page 6: "From the results findings, it shows that there are three main themes..."
- Page 2: "Limitations include a small sample size and brief data collection period..."

**Suggestions for Improvement**:

- Rephrase page 6 to: "Thematic analysis revealed three primary themes: understanding of aviation English, content integration, and instructional challenges."
- On page 2, suggest: "Future studies should expand sample size and data collection timeframe."
- On page 12, rephrase to: "Teachers acquire aviation knowledge through iterative processes, supporting enhanced training programs."
- On page 13, add: "Future mixed-methods studies could quantify theme prevalence."

Is the work clearly and accurately presented and does it cite the current literature?

Yes

If applicable, is the statistical analysis and its interpretation appropriate?

Not applicable

Are all the source data underlying the results available to ensure full reproducibility?

Yes

Is the study design appropriate and is the work technically sound?

Yes

Are the conclusions drawn adequately supported by the results?

Yes

Are sufficient details of methods and analysis provided to allow replication by others?

Yes

Reviewer Expertise:

The article presents a solid exploratory qualitative study on the experiences of Malaysian aviation English teachers, with a clear structure, appropriate methods (semi-structured interviews and thematic analysis), and conclusions supported by the data. The small sample size (n=3) is justified for in-depth insights, and trustworthiness measures like member checking and external audits enhance rigor. However, reservations stem from minor but addressable issues: the literature review could incorporate more recent 2024-2025 citations on post-pandemic ESP adaptations in aviation for greater currency; some sentences need rephrasing for clearer attribution and academic tone; the small sample justification could reference qualitative norms; and expanded details on methods (e.g., interview mode preferences) would improve replicability. These specific revisions would elevate the article's merit without requiring major overhauls.

I confirm that I have read this submission and believe that I have an appropriate level of expertise to confirm that it is of an acceptable scientific standard.
